# A cross-sectional study of self-reported general health, lifestyle factors, and disease: the Hordaland Health Study

**DOI:** 10.7717/peerj.609

**Published:** 2014-10-02

**Authors:** Randi Jepsen, Tadesse Washo Dogisso, Elin Dysvik, John Roger Andersen, Gerd Karin Natvig

**Affiliations:** 1 Department of Global Public Health and Primary Care, University of Bergen, Bergen, Norway; 2 Faculty of Health Studies, Sogn og Fjordane University College, Førde, Norway; 3 Department of Health Studies, University of Stavanger, Stavanger, Norway; 4 Førde Health Trust, Førde, Norway

**Keywords:** Life style, Epidemiology, Self report, Health, Obesity, Physical activity, Smoking, Alcohol

## Abstract

**Background.** Information on self-reported health is important for health professionals, and the aim of this study was to examine associations between lifestyle factors and self-reported health and the mediating effect of disease in a Norwegian population.

**Methods and Materials.** The data collection was conducted as part of the Hordaland Health Study (HUSK) 1997–99, which was a cross-sectional epidemiological study. All individuals in Hordaland county born in 1953–1957 were invited to participate (aged 40–44 years). Complete information for the present study was obtained from 12,883 individuals (44% response rate). Height and weight were measured at a physical examination. Information on lifestyle factors, self-reported health, disease (heart attack, apoplexy, angina pectoris, and diabetes), and socio-demographic variables was obtained from a self-administered questionnaire. Self-reported health was measured with a one-item question. Odds ratios for fair or poor self-reported health were calculated using multiple logistic regression analyses adjusted for disease and socio-demographic variables.

**Results.** Respondents reporting adverse lifestyle behaviours (obesity (odds ratio (OR) 1.7, *p* < 0.001), smoking (OR 1.2, *p* < 0.001), or excessive intake of alcohol (OR 3.3, *p* < 0.001)) showed an increased risk of poor self-reported health. Furthermore, a moderate intake of wine (OR 0.6, *p* < 0.001) or strenuous physical activity (OR 0.5, *p* < 0.001) decreased the risk of poor health. Disease did not mediate the effect.

**Conclusion.** A one-item question measuring self-reported health may be a suitable measure for health professionals to identify levels of subjective health and reveal a need to target lifestyle factors in relatively young individuals with or without disease.

## Background

Patient-reported information has been acknowledged over the past decades as measures to capture subjective perspectives and inform and improve clinical practice. One of these measures is self-reported health (Ahmed et al., 2012). Jylha (2009) has proposed a conceptual model to help identifying the different types of information on which people base their health assessments. In this model, the evaluation of one’s own health encompasses a review of information, such as functional status, diseases, health-related behaviours, and socio-demographic factors. Additionally, the model consists of factors comprising contextual frameworks, such as cultural conventions. As an example of the significance of self-reported health, Idler & Benyamini (1997) reported that it predicted mortality in almost all of the 27 community studies they included in their review, and this connection may be seen as well established. However, the question of how people may understand and interpret the concept of self-reported health is not fully unravelled empirically.

Lifestyle factors are among the behaviours that individuals consider when they report their own general health (Manderbacka, 1998; Jylha, 2009) and previous research found that cigarette smoking and alcohol consumption were related to reduced physical and mental health (Riise, Moen & Nortvedt, 2003). In addition, obesity has been found as a predictor of self-rated health (Prosper, Moczulski & Qureshi, 2009). The experience of general health has also been found to be positively associated with levels of physical activity (Malmberg et al., 2005). One study, including several lifestyle factors, found a negative relationship between unhealthy lifestyle and mental and physical health (Pisinger et al., 2009). Another study (Manderbacka, Lundberg & Martikainen, 1999) examined whether different risk factors contributed to self-reported health, when the effect of various health problems were also included. In this study, the authors examined the relationship between risk factors, such as dietary habits, exercise, smoking, alcohol consumption, and body mass index (BMI), and self-reported health. The authors found that these associations were weakened or not significant at all when they were adjusted for health problems. However, these health problem variables were composed by, on the one hand, subjective phenomena including symptoms and functional problems, which may overlap the concept of self-reported health, and, on the other hand, objective medical diagnoses. Thus, the specific significance of the diseases was not revealed. Therefore, the aim of the present study was to examine associations between various lifestyle factors and self-reported health and the mediating effect of disease. The research question guiding the study was if lifestyle factors (smoking, alcohol consumption, leisure time physical activity, and BMI) worked as risk factors for self-reported general health in an adult Norwegian population, with adjustment for socio-demographic characteristics (gender, marital status, and educational level) and diseases (coronary heart disease, apoplexy, and diabetes).

## Methods and Materials

### Design and sample

The Hordaland Health Study (HUSK) was conducted during 1997–99 as a collaboration between the National Health Screening Service, the University of Bergen, and local health services. The study design was cross-sectional. The study population included all individuals in Hordaland county born in 1953–1957 (29,400). A total of 8,584 men and 9,976 women participated, yielding a participation rate of 57% for men and 70% for women. Complete information of all variables included in the present study was available for 12,883 individuals (44% of the study population).

### Ethical approval

Written informed consent was obtained from each participant prior to the study. The study protocol was approved by the Regional Committee for Medical Research Ethics, Health Region III (reference number 311/97-64.97) and the Norwegian Data Inspectorate (reference number 97/1504-2). The study conforms to the Declaration of Helsinki.

### Measures

Information on self-rated health, lifestyle factors, diseases, and socio-demographic variables were obtained from a self-administered questionnaire which was sent by mail together with a letter of invitation and the time of appointment for a physical examination. The questionnaire was collected and height and weight were measured at the examination (Naess et al., 2008).

Self-rated health was measured by one question: “How is your health at the moment?” with four response categories: “poor”, “fair”, “good”, and “very good”. In their review of community studies, Idler & Benyamini (1997) found that one-item measures on self-reported health revealed significant, independent associations with mortality. The Norwegian measure used in this study has previously shown predictive value for morbidity (Naess et al., 2005; Riise et al., 2014) and mortality (Dalen et al., 2012).

The measures of lifestyle were leisure time physical activity, tobacco smoking, alcohol consumption, and BMI. Physical activity was assessed by the Cohort of Norway (CONOR) instrument which comprises two questions about the average weekly number of hours of either light (not sweaty or short of breath) or strenuous leisure time physical activity the last year (Graff-Iversen et al., 2008). The categories are “none”, “less than 1 h”, “1–2 h”, or “3 h or more” for both questions. Validity of this CONOR instrument has been demonstrated through correlation with anthropometric and biological variables in epidemiological research (Aires, Selmer & Thelle, 2003; Graff-Iversen et al., 2008). Data on tobacco use were obtained from questions about current and former smoking habits. This measure has shown predictive validity for the risk of coronary heart disease (Igland et al., 2012). Alcohol concumption was measured with items about the prevalence of consumption of wine, beer, and liquor within the last two weeks. This instrument has shown predictive validity in epidemiological research (Torvik, Rognmo & Tambs, 2012).

BMI was calculated as kilograms per square meter and categorised in accordance with the World Health Organization classification of underweight (<18.5 kg/m^2^), normal weight (18.5–24.9), overweight (25–29.9), obesity grade I (30–34.9), and obesity grade II and more (35 +) (World Health Organization, 1998).

### Statistical analyses

The responses on self-reported health were dichotomized into “very good/good” and “fair/poor”. Marital status was dichotomized into “married/cohabiting” or “other”. Education was used as an indicator of socioeconomic status and the five categories for educational level were coded as “low” (up to and including 10 years of schooling), “medium” (high school), and “high” (college/university). A dichotomous variable of “not having a disease” or “having one or more diseases” was coded from self-reported occurence of heart attack, apoplexy, angina pectoris, and diabetes. Data on tobacco smoking were coded into three categories; “never a smoker”, “formerly a smoker” (without specification of time since cessation), or “currently a smoker”. Units of alcohol per two weeks were catogorized as “none”, “1–14”, or “15 or more” for wine, beer, and liquor, respectively. 15 units/two weeks have previously been used as a cut-off point for high alcohol consumption (Myrtveit et al., 2013).

Descriptive statistics were used to present the sample. Univariate and multivariate logistic regression were applied to study associations between lifestyle factors (consumption of wine, beer, and liquor; light and strenuous physical activity; smoking; and BMI) and self-rated health. Disease, gender, marital status, and educational level were included as covariates. The age range was very small, and preliminary analysis showed that it did not influence the results (data not shown). Therefore, it was not included in the analysis. A *p*-value ≤0.05 indicated statistical significance. The statistical package IBM SPSS for Windows, version 20.0, was used in the analyses.

## Results

### Study sample

All study participants (12,883) were aged 40–44 years. Among these, 51.4% were women (Table 1). A smaller proportion (11.5%) had a BMI of 30 or more, i.e., obesity, while 49.5% were of normal weight. The most excessive alcohol consumption was reported by 1% of the participants for wine, 2.2% for beer and 0.2% for liquor. Forty-three percent reported doing light physical activity and 13.1% strenuous physical activity for three hours or more per week. Current smoking was reported by 34.4% of the participants. Currently having or having experienced coronary heart disease, apoplexy, and/or diabetes were reported by 9.8% (Table 1).

**Table 1:** Characteristics of the participants (*N* = 12,883).

Variables	%
**Self-rated health**	
Very good/good	87.0
Fair/poor	13.0
**Gender**	
Male	48.6
Female	51.4
**Marital status**	
Married/cohabiting	74.6
Other	25.4
**Education**	
Low	17.0
Medium	45.8
High	37.2
**Disease (coronary heart disease, apoplexy, or diabetes)**	
No	90.2
Yes	9.8
**Body mass index**	
<18.5	0.8
18.5–24.9	49.5
25–29.9	38.8
30–34.9	9.1
35+	2.4
**Wine (units per two weeks)**	
None	50.7
1–14	48.3
15+	1.0
**Beer (units per two weeks)**	
None	54.2
1–14	43.6
15+	2.2
Liquor (units per two weeks)	
None	79.1
1–14	20.8
15+	0.2
**Light physical activity (hours per week)**	
None	4.3
<1	14.6
1–2	38.1
3+	43.0
**Strenuous physical activity (hours per week)**	
None	29.7
<1	28.8
1–2	28.4
3+	13.1
**Smoking**	
Never smoker	37.6
Former smoker	28.0
Current smoker	34.4

Female gender and not being married/cohabiting were related to higher odds for fair or poor health. Favourable self-reported health increased with level of education (Table 2).

**Table 2:** Odds ratio (OR) with 95% confidence intervals (CI) for fair or poor self-rated health (*N* = 12,883).

	Univariate model			Multivariate model		
Variables	OR	95% CI	*p*-value	OR	95% CI	*p*-value
**Gender**						
Male (ref)	1			1		
Female	1.4	1.2, 1.5	<0.001	1.3	1.2, 1.5	<0.001
**Marital status**						
Married/cohabiting (ref)	1			1		
Other	1.5	1.4, 1.8	<0.001	1.5	1.4, 1.7	<0.001
**Education**						
Low (ref)	1			1		
Medium	0.6	0.5, 0.7	<0.001	0.7	0.6, 0.8	<0.001
High	04	0.4, 0.5	<0.001	0.6	0.5, 0.7	<0.001
**Disease**						
No (ref)	1			1		
1+	2.9	2.5, 3.3	<0.001	2.7	2.3, 3.1	<0.001
**Body mass index**						
<18.5	1.5	0.9, 2.5	0.149	1.2	0.7, 2.0	0.556
18.5–24.9 (ref)	1			1		
25–29.9	1.1	0.9, 1.1	0.422	1.1	1.0, 1.3	0.089
30–34.9	1.9	1.6, 2.1	<0.001	1.6	1.3, 1.9	<0.001
35+	2.3	1.8, 3.1	<0.001	1.7	1.3, 2.3	<0.001
**Wine (units per two weeks)**						
None (ref)	1			1		
1–14	0.5	0.5, 0.6	<0.001	0.7	0.6, 0.8	<0.001
15+	0.6	0.5, 1.1	0.113	0.8	0.4, 1.4	0.421
**Beer (units per two weeks)**						
None (ref)	1			1		
1–14	0.6	0.6, 0.7	<0.001	0.8	0.7, 0.9	0.001
15+	1.1	0.8, 1.5	0.604	1.1	0.8, 1.5	0.642
**Liquor (units per two weeks)**						
None (ref)	1			1		
1–14	0.8	0.7, 0.9	<0.001	1.0	0.9, 1.2	0.916
15+	4.9	2.2, 11.3	<0.001	3.3	1.4, 7.9	<0.001
**Light physical activity (hours per week)**						
None (ref)	1			1		
<1	0.8	0.6, 1.0	0.030	0.9	0.7, 1.2	0.576
1–2	0.5	0.4, 0.6	<0.001	0.7	0.6, 0.9	0.008
3+	0.5	0.4, 0.6	<0.001	0.7	0.6, 0.9	0.010
**Strenuous physical activity (hours per week)**						
None (ref)	1			1		
<1	0.6	0.5, 0.7	<0.001	0.8	0.7, 0.9	<0.001
1–2	0.5	0.4, 0.5	<0.001	0.7	0.6, 0.8	<0.001
3+	0.4	0.3, 0.5	<0.001	0.5	0.4, 0.7	<0.001
**Smoking**						
Never smoker (ref)	1			1		
Former smoker	0.9	0.8, 1.0	0.030	0.9	0.8, 1.0	0.052
Current smoker	1.4	1.2, 1.6	<0.001	1.2	1.1, 1.4	<0.001

#### Lifestyle factors and self-rated health

As seen in Table 1, 87% of the participants rated their health as very good or good, and 13% as fair or poor. In Table 2, the odds of rating health as fair or poor are shown. Among the lifestyle-related variables, obesity was significantly related to an increased risk of fair or poor health. Being a current smoker also increased the odds ratio of fair or poor health whereas the unadjusted analysis—but not the adjusted—revealed better health in former smokers than in participants who never smoked. In addition, the consumption of 15 or more units of liquor per two weeks was significantly related to adverse self-reported health. On the other hand, a moderate intake of wine or beer (1–14 units per two weeks) decreased the odds ratio of fair or poor health. The same applied to both light and strenuous physical activity. The associations between self-reported health and lifestyle factors were similar, whether socio-demographic variables or the disease variable were included into the statistical model or not. Hence, we did not find a mediating effect of the disease variable.

## Discussion

In this population study we found that the risk of adverse self-reported health was related to obesity, current smoking, and consumption of 15 or more units of liquor per two weeks. On the other hand, a moderate intake of wine or beer and leisure time physical activity decreased the risk of unfavourable self-reported health.

Self-reported health, as measured by a one-item scale, has previously been well established as a predictor of mortality (Idler & Benyamini, 1997; Dalen et al., 2012) and previous studies also found that self-reported health was a predictor of diabetes (Naess et al., 2005) and lung cancer (Riise et al., 2014). The question of what kind of information each individual bases his or her evaluation on when answering this question, is previously discussed and encompasses a wide range of factors of both individual and contextual character. Among these factors are lifestyle behaviours (Manderbacka, 1998; Jylha, 2009).

Overweight and obesity have a broad spectrum of individual, social, and environmental explanatory factors, and can also be seen as lifestyle-related health problems (World Health Organization, 1998). Furthermore, it is a rising public health and clinical problem (Fruhbeck et al., 2013) and data from the Norwegian population show that the levels of body weight have increased during the recent decades (Midthjell et al., 2013). The prevalence of overweight among children and adolescents is high too (Dupuy et al., 2011). As in the present study, Prosper and colleagues found that high levels of BMI were predictors of adverse self-reported health (Prosper, Moczulski & Qureshi, 2009). Similar findings are reported with health-related quality of life (Soltoft, Hammer & Kragh, 2009). Previous research has also shown that high levels of BMI are associated with cardiovascular risk (Jonsson et al., 2002) and mortality (Flegal et al., 2013).

One of the main changes in health behaviours in the past years has been a decrease in physical activity both at work and at leisure, and the rise in overweight and obesity is often connected to this problem (Anderssen et al., 2008). One recent study found that obese participants had lower overall physical activity compared to normal weight participants (Hansen et al., 2013). Physical activity can also be positively associated with self-reported health, irrespective of an increased BMI (Herman et al., 2012). We found that both light and strenuous physical activity decreased the risk of fair or poor health. A similar finding is reported previously (Tran et al., 2013), and daily walking has been found to be inversely related to mortality among elderly people (Samawi, 2013).

We found that the consumption of 15 or more units of liquor per two weeks increased the odds ratio of fair or poor self-reported health, and this result is in accordance with previous research on binge drinking (Tsai et al., 2010). The results regarding a decreased risk of adverse health for individuals with a moderate intake of wine or beer, are more inconsistent. In examining the association between harmful patterns of alcohol consumption and self-reported health status measured by Euro-QoL Group’s five-dimension questionnaire (EQ5D), Petrie et al. (2008) found that all levels of risky alcohol use, including the low risk level, were associated with lower self-ratings of health. There was, however, a small effect for those with a moderate alcohol intake. On the other hand, previous studies, examining the association between moderate intake of alcohol and diseases, found that a moderate intake of alcohol, including red wine, reduced the risk of cardiovascular, cerebrovascular, and peripheral vascular diseases in populations (Szmitko & Verma, 2005), and one recent study confirmed these results, showing that moderate alcohol consumption was associated with lower risk of stroke in a population of women (Jimenez et al., 2012). Furthermore, our result, showing that smoking was associated with poor health, is supporting previous studies regarding self-reported health (Goldman, Glei & Chang, 2004; Tran et al., 2013) as well as health-related quality of life (Strine et al., 2008). Although the odds ratio was small, it was unexpected that the unadjusted analysis revealed better health in former smokers than in study participants who never smoked. Assocations between smoking cessation and positive affect (Branstrom et al., 2010) or mental health (Taylor et al., 2014) may serve as an explanation. However, Manderbacka, Lundberg & Martikainen (1999) and Kaleta et al. (2006) reported opposite results whereas Ho et al. (2003) found no statistical difference in self-rated health between former and never smokers. None of the studies, including the present, took time since smoking cessation into consideration.

Our finding that the disease variable did not mediate the effect of lifestyle factors on self-reported health differs from the results reported by Manderbacka, Lundberg & Martikainen (1999). However, as mentioned, they included not only medical diagnoses, but also a range of illnesses, functional problems, and symptoms in their operationalisation of health problems, leaving the question of the role of diseases unanswered.

In line with other studies (Eriksson, Unden & Elofsson, 2001; Nielsen et al., 2008), we found that female gender was associated with fair or poor health. The same counted for living alone. Joung et al. (1995) found that married men and women reported better health than unmarried, partly independent of lifestyle factors. Helweg-Larsen, Kjøller & Thoning (2003) found higher mortality in unmarried than married individuals. The positive association between length of education and self-rated health found in the present study was also reported by Mackenbach et al. (2008) and Liu & Hummer (2008).

The study population was aged 40–44 years which may limit the generalisability of our findings. Except for type 1 diabetes (Tao et al., 2010), the incidence of the medical conditions included in the disease variable of our study increases with age (World Health Organization, 2011). The impact of disease on self-rated health may vary between age groups. There was no information about the severity of the diseases we included in the disease variable, which may have influenced the results of our study.

The data collection was performed in 1997–99. Hence, the study provides knowledge from a time when one third of Norwegian adults were daily smokers, compared to the current situation where less than one in five smokes tobacco on a daily basis (Norwegian Institute of Public Health, 2014b), purchase of alcohol was lower (Norwegian Institute of Public Health, 2014a), physical activity levels were higher (Anderssen et al., 2008), and the proportion of individuals with overweight and obesity was at a rise (Midthjell et al., 2013). Changed patterns of lifestyle may affect cultural conventions which, according to Jylha (2009), play a role in subjective assessments of health.

### Methodological considerations

The data material from HUSK is based on a large sample which gave statistical power to include a number of variables in the multivariate analysis. Similar to other epidemiological surveys (Sogaard et al., 2004), the response rate was rather low (44%). Whether this created bias in the results is not possible to determine. However, some studies indicate that non-participation may not impact very much on associations between study variables in large health surveys (Sogaard et al., 2004; Laaksonen et al., 2008). The data on height and weight were measured by clinicians whereby we avoided the under-reporting for weight and over-reporting for height that are common in self-reports (Connor Gorber et al., 2007). Due to the cross-sectional design of the study we cannot infer causal relationships.

## Conclusion

A wide variety of factors are included when individuals are reporting how they evaluate their current health. Our study adds to the body of empirical knowledge, showing associations between unhealthy lifestyle and self-reported health which are not mediated by diseases in adults aged 40–44 years. As a supplement or alternative to health assessments and interpretations done by health professionals, the one-item question may be suitable for health professionals as an instrument to identify how patients or other recipients of health services evaluate their health and an indicator of when lifestyle should be targeted in relatively young individuals with or without disease.
